# Supplementary material for: Integrating Serum Biomarkers into Prediction Models for Biochemical Recurrence Following Radical Prostatectomy
Source: Cancers (Basel). 2021 Aug 19;13(16):4162. doi: 10.3390/cancers13164162 (PMC8391749; doi:10.3390/cancers13164162)
Supplement: Supplementary file 1 [file cancers-13-04162-s001.zip › cancers-1321429-supplementary.pdf]

cancers

MDPI

Supplementary Materials: Integrating Serum Biomarkers into Prediction Models for Biochemical Recurrence following Radical Prostatectomy

Shirin Moghaddam, Amirhossein Jalali, Amanda O'Neill, Lisa Murphy, Laura Gorman, Anne-Marie Reilly, Áine Heffernan, Thomas Lynch, Richard Power, Kieran J. O'Malley, Kristin A. Taskèn, Viktor Berge, Vivi-Ann Solhaug, Helmut Klocker, Brendan Murphy and R. William Watson

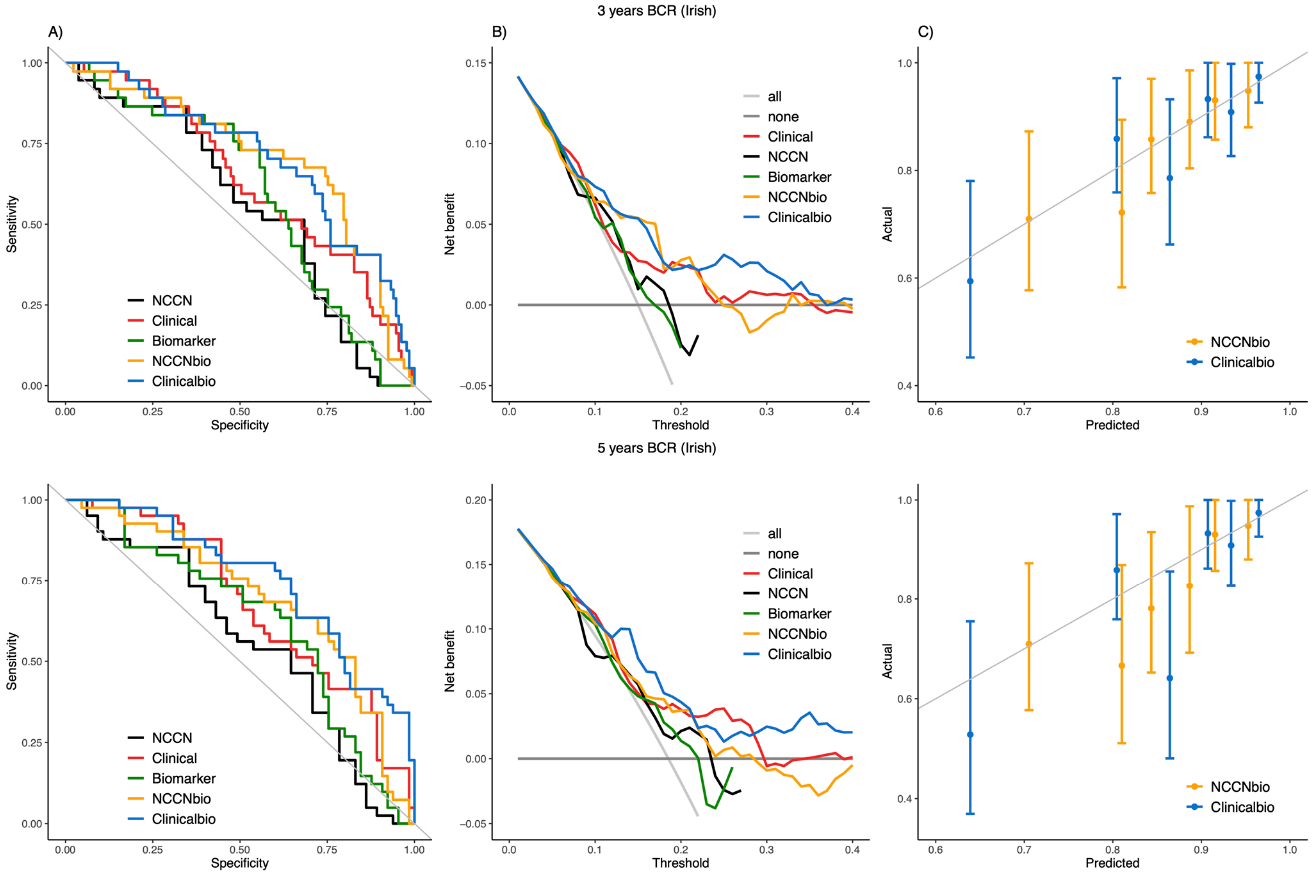

Figure S1. ROC curve (**A**), decision curve (**B**) and calibration plot (**C**) of the 'NCCNbio' and 'Clinicalbio' models internally compared with 'NCCN', 'Clinical' and 'Biomarker' models in the Irish cohort at 3-year (on the top) and 5-year (on the bottom).

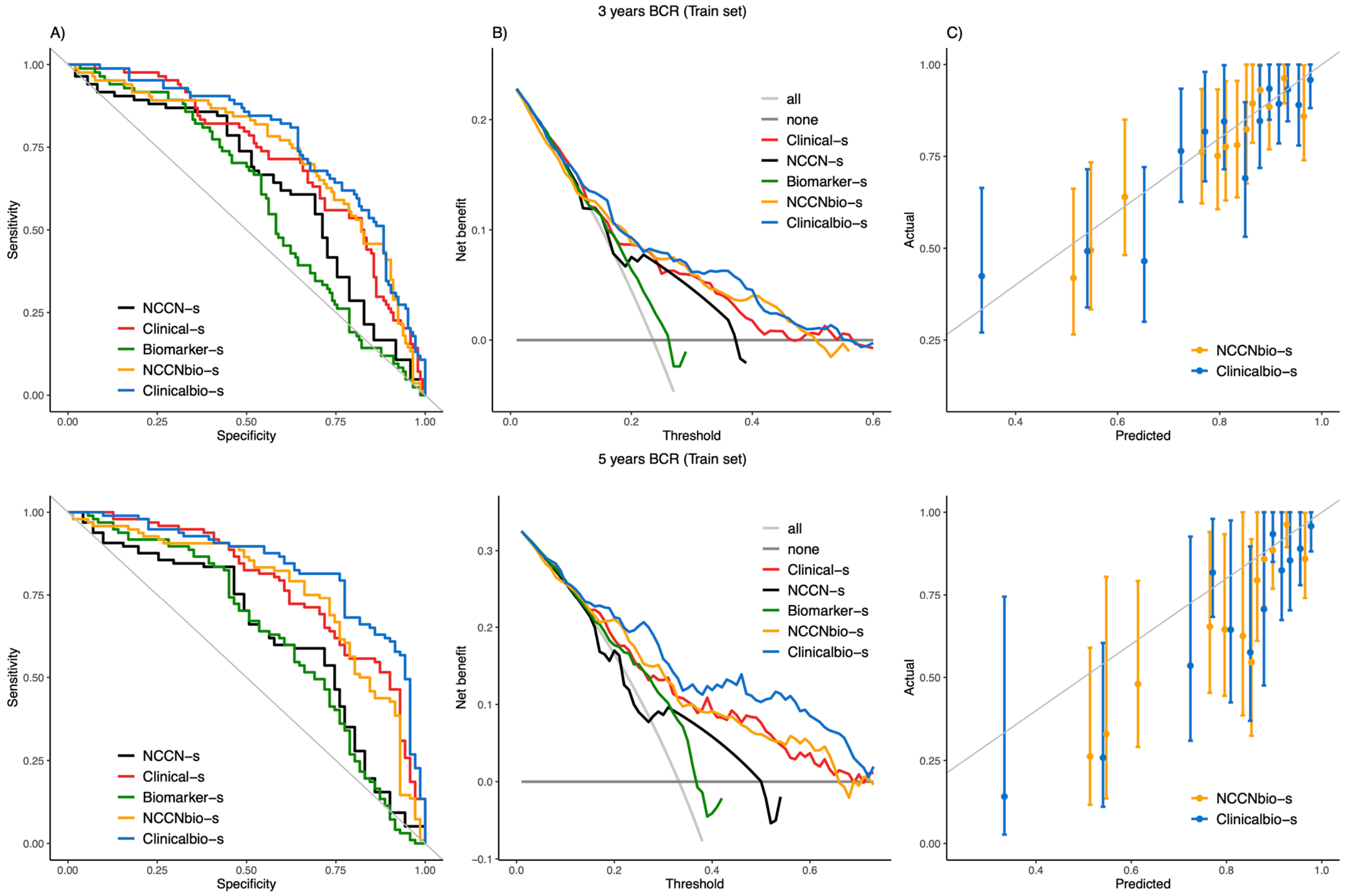

Figure S2. ROC curve (**A**), decision curve (**B**) and calibration plot (**C**) of the 'NCCNbio-s' and 'Clinicalbio-s' models internally compared with 'NCCN', 'Clinical' and 'Biomarker' models in the train set at 3-year (on the top) and 5-year (on the bottom).

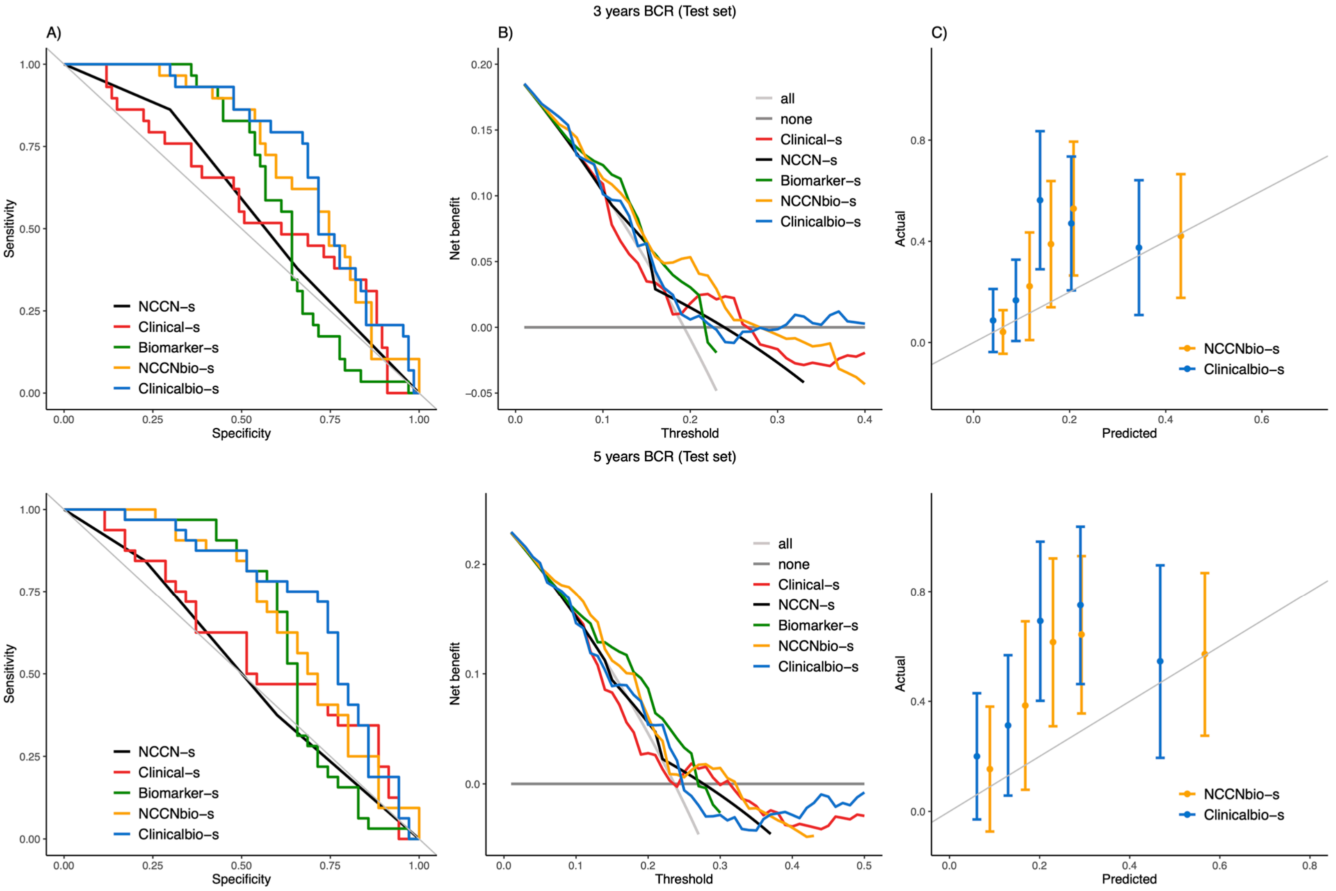

Figure S3. ROC curve (**A**), decision curve (**B**) and calibration plot (**C**) of the 'NCCNbio-s' and 'Clinicalbio-s' models externally compared with 'NCCN', 'Clinical' and 'Biomarker' models in independent test set at 3-year (on the top) and 5-year (on the bottom).

Table S1. Clinical characteristics of the patients in the train and test sets including the univariate *p*-value for each clinical variable [a].

Features	Train set	Test Set	*p*-Value[b]
**Sample size (*n* = 577)**	405	172	
**Cohort samples**			
Irish	184 (45%)	87 (51%)	
Austrian	90 (22%)	38 (22%)	
Norwegian	131 (32%)	47 (27%)	
**Pre-op**			
PSA			0.8
Mean (SD)	8.77 (6.18)	8.54 (5.47)	
DRE			0.9
Normal	331 (82%)	139 (81%)	
Abnormal	74 (18%)	33 (19%)	
Biopsy Gleason Score			0.2
6	183 (45%)	80 (46%)	
7	147 (36%)	70 (41%)	
8 and above	75 (19%)	22 (13%)	
**Post-op**			
Gleason Score			0.004
6	121 (30%)	68 (40%)	
7	192 (48%)	81 (48%)	

8 and above	91 (22%)	20 (12%)	
Stage			0.4
Organ confined	221 (55%)	101 (59%)	
Non organ confined	184 (45%)	71 (41%)	
**Time to biochemical reoccurrence**			0.2
< 3 years	23.6 %	19.3 %	
< 5 years	33.2 %	23.7 %	

[a] Statistics presented: median (IQR); *n* (%), [b] Statistical tests performed: Kruskal-Wallis test; chi-square test of independence; log-rank test.

Table S2. Summary of the 'NCCNbio-s' and 'Clinicalbio-s' models developed on the train set using hazard ratio, 95% confidence interval (CI) for hazard ratio and *p*-value for each risk factor in the model.

Features	**NCCNbio-s**			**Clinicalbio-s**		
	**Hazard Ratio**	**95% CI**	***p*-Value**	**Hazard Ratio**	**95% CI**	***p*-Value**
PSA[a]	-	-	-	2.616	[1.87, 3.66]	< 0.001
DRE						
(Abnormal vs normal)	-	-	-	1.47	[0.90, 2.40]	0.124
Biopsy Gleason Score						
(7 vs 6)	-	-	-	1.597	[0.95, 2.69]	0.079
(8 or above vs 6)	-	-	-	3.239	[1.89, 5.56]	< 0.001
NCCN						
(Intermediate vs low)	1.51	[0.82, 2.77]	0.183	-	-	-
(High vs low)	4.18	[2.47, 7.07]	< 0.001	-	-	-
APOAII[¥] (pg/ml)	1.26	[0.93, 1.71]	0.139	-	-	-
ZAG[¥] (pg/ml)	0.79	[0.56, 1.11]	0.179	-	-	-
PEDF (100 000 pg/ml)	0.735	[0.61, 0.88]	< 0.001	0.696	[0.58, 0.84]	< 0.001

[a] The non-linear effect of the predictor using a log transformation.

Table S3. The AUC for the 'NCCNbio-s' and 'Clinicalbio-s' compared to 'NCCN-s', 'Clinical-s' and 'Biomarker-s' for internal validation using the Train set (panel A) and external validation using the Test set (panel B).

**Models**	**A) Internal Validation**		**B) External Validation**	
	AUC at 3-year (Train set)	AUC at 5-year (Train set)	AUC at 3-year (Test set)	AUC at 3-year (Test set)
NCCN-s	0.6241	0.6218	0.5654	0.5121
Clinical-s	0.7218	0.7778	0.5816	0.5848
Biomarker-s	0.5907	0.631	0.6186	0.6438
NCCNbio-s	0.7329; *p*-value (vs NCCN-s) = 0.037 [a]	0.7601; *p*-value (vs NCCN-s) = 0.020 [a]	0.7102; *p*-value (vs NCCN-s) < 0.001 [a]	0.6848; *p*-value (vs NCCN-s) = 0.001 [a]
Clinicalbio-s	0.7713; *p*-value (vs Clinical-s) = 0.013 [a]	0.8332; *p*-value (vs Clinical-s) = 0.023 [a]	0.7360; *p*-value (vs Clinical-s) = 0.015 [a]	0.7330; *p*-value (vs Clinical-s) = 0.042 [a]

[a] DeLong test *p*-value.
